# IL-1β and CXCR4 as Potential Therapeutic Targets for Alzheimer’s Disease

**DOI:** 10.2174/0113862073295516240508173238

**Published:** 2024-05-20

**Authors:** Yuhan Zhang, Qiong Su, Jun Tan, Weiping Deng, Xiaoju Wan, Yunjun Li, Kuanyong Shu

**Affiliations:** 1 Department of Reproduction Center, Jiangxi Maternal and Child Health Hospital, Nanchang, Jiangxi, China;; 2 Department of Oncology, Jiangxi Maternal and Child Health Hospital, Nanchang, Jiangxi, China

**Keywords:** Alzheimer’s *disease*, bioinformatics, differentially expressed genes, LASSO, biomarkers, protein-protein interaction

## Abstract

**Background:**

Alzheimer’s Disease (AD) is a highly prevalent form of age-related dementia. However, the underlying mechanisms of AD are largely unexplored.

**Materials and Methods:**

In this study, bioinformatics analysis was performed to identify the possible therapeutic targets for AD. The GEO database was used to screen the Differentially Expressed Genes (DEGs). Enrichment analysis, protein-protein interaction network, and LASSO model analyses were successfully performed. Furthermore, an ELISA assay was also conducted to determine the expression of principal genes within the AD and control samples.

**Results:**

A total of 416 differentially expressed genes (DEGs) were recognized based on the GSE48350 and GSE28146 datasets. The IL-1β and CXCR4 levels were markedly elevated in the AD samples relative to the control.

**Conclusion:**

The IL-1β and CXCR4 genes were identified as principal AD-related genes that can be targeted for anti-AD therapy.

## 1 INTRODUCTION

Alzheimer’s Disease (AD) is a highly degenerative neurological disorder, in which the patient suffers from tremendous memory deficits [1-4]. Based on several *epidemiological studies,* AD currently affects over 47 million people worldwide [5-7]. The pathophysiology of AD (particularly amyloid plaques and neurofibrillary tangles) and its genetic drivers have been extensively explored recently [8-12]. However, thus far, *the precise* pathogenesis *of* AD *remains unknown*, and no effective prevention and/or treatment methods exist for AD [13]. Thus, specific therapeutic targets for AD are urgently needed.

The *GEO* (Gene Expression Omnibus) database consists of microarray, sequencing, and high-throughput functional genomic datasets [14-17]. Our goal was to search for hub genes and potential molecular mechanisms related to AD using bioinformatics. To that end, we retrieved AD *versus* normal tissue microarray data from the GEO databases and performed enrichment, protein-protein interaction (PPI) network, as well as LASSO regression analyses to screen for principal AD-related genes, which were verified using ELISA assay. Hence, we provided a basis for the generation of new highly efficacious anti-AD drugs that can specifically target the underlying mechanism(s) of AD pathogenesis.

## 2 MATERIALS AND METHODS

### 2.1 Microarray Information

We retrieved data from the GEO database (GSE48350 and GSE28146). The GSE28146 dataset comprised 22 AD CA1 tissues and 8 healthy control samples, whereas the GSE48350 database contained 80 AD CA1 tissues and 173 healthy control samples. Both datasets were based on the GPL570 platform.

### 2.2 Data Processing

The raw GSE48350 and GSE28146 datasets were preprocessed using R (version 4.0.2). *P <0.05 and |log2 Fold Change| >1* were employed for confirmation of the DEGs in AD [18]. DEGs were further processed and plotted into volcano plots using ggplot2.

### 2.3 Enrichment Analysis of DEGs

Metascape [19-21] is a comprehensive software for gene annotation and enrichment analysis. Gene enrichment was analysed from GO [22-24] and KEGG [25-27] through the database to predict the potential biological value of DEGs.

### 2.4 Construction of the PPI Network

STRING (https://string-db.org/) was employed for the construction of the Protein-Protein Interaction (PPI) networks [28]. A confidence score of ≥0.4 was set as the threshold. Cytoscape software (version 3.7.2) and the CytoHubba plugin (version 0.1) were employed for the visualization and identification of the PPI network. The filtering algorithm was then used to determine the 20 highest-ranking hub genes based on Closeness, Stress, EcCentricity, and Degree. Lastly, the Venn diagram validated the significance of the principal genes in AD.

### 2.5 Construction of the LASSO Model

Using the newly identified principal genes, we constructed a model of Least Absolute Shrinkage and Selection Operator (LASSO) because of its strong predictive value using the glmnet package (http://www.bioconductor.org/packages/glmnet/) [29-31].

### 2.6 Animals

All animal protocols abided according to the National Institutes of Health (NIH) guidelines and received approval from the Nanchang University Animal Care and Use Committee. Ten adult (3 months) and ten elderly (18 months) male C57BL/6 mice were obtained from the Hunan Slake Jingda Laboratory Animal Co., Ltd, Hunan, China. They received a standard diet and free drinking water for over 4 weeks prior to the start of the experiments. After four weeks, the mice were anesthetized with 4% isoflurane, intubated, and the hippocampal tissues were extracted.

### 2.7 ELISA Assays

Hippocampal tissue lysates were homogenized in PBS and stored overnight at -20°C for the ELISA assay. The following day, the homogenates underwent centrifugation at 4,000 g for 10 min, and the supernatants were placed into a new tube. The following ELISA kits (IL-1β, CXCR4, and TAC1) were next employed for the measurement of protein levels in samples, as per kit directions (Jiangsu Meimian Industrial Co., Ltd, Jiangsu, China).

### 2.8 Statistical Analysis

All experiments were performed thrice and expressed as mean± SD. All statistical analyses were conducted with the Prism software (version 8, GraphPad). Inter-group comparisons were made with an unpaired t-test. *P* <0.05 was defined as significant.

## 3 RESULTS

### 3.1 DEG Analysis

**Fig. (1):**
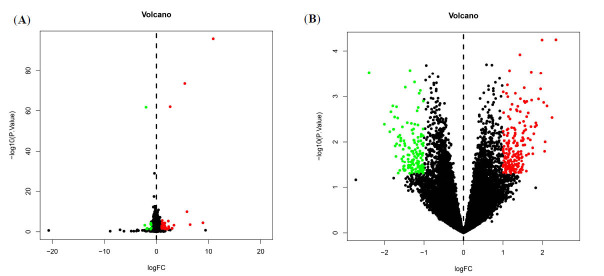
Differentially Expressed Genes (DEGs) identified among Alzheimer’s *disease* (AD) and control samples. (**a-b**) Volcano plot of GSE48350 and GSE28146; Red dots denote upregulated genes; Green dots denote downregulated genes.

Overall, the microarray data from 101 *AD patients and* 181 *normal* subjects were found suitable for this study. A total of 416 DEGs were recognized with criteria of *P at <0.05 and |log2 Fold Change| >1* (Fig. **1**).

### 3.2 Enrichment Analysis of DEGs

**Fig. (2):**
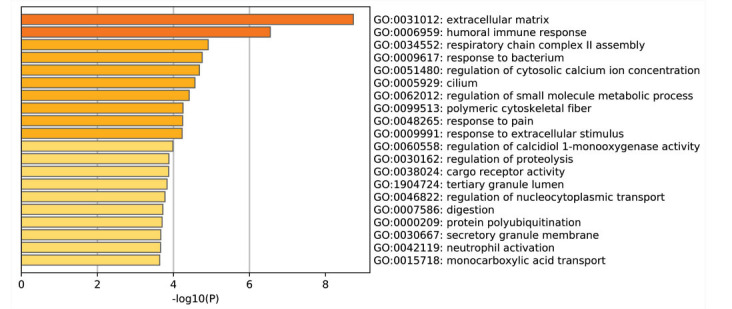
Biological functions based on Gene Ontology (GO) analysis of the Differentially Expressed Genes (DEGs).

**Fig. (3):**
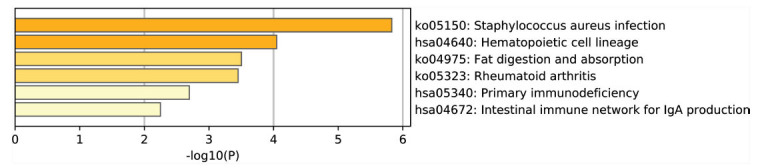
KEGG pathway analysis of Differentially Expressed Genes (DEGs).

The GO enrichment analysis was carried out using the Metascape software. GO functional analysis was categorized into 3 groups, namely BP, CC, and MF. Based on our analysis, DEGs were primarily enriched in the ‘Extracellular space’, ‘Calcium-dependent protein binding’, and ‘Respiratory chain complex II assembly’ (Table **1** and Fig. **2**). DEGs pathway enrichment analysis with KEGG further revealed that it was primarily enriched in ‘staphylococcus aureus infection’, ‘hematopoietic cell lineage’, and ‘fat digestion and absorption’ (Table **2** and Fig. **3**).

### 3.3 PPI Network Generation

**Fig. (4):**
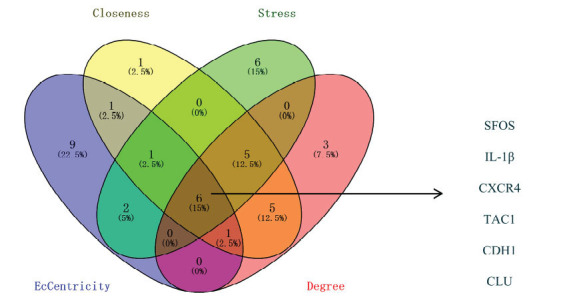
Common genes, based on closeness, stress, eccentricity, and degree algorithm.

The PPI network was generated using the STRING database in Cytoscape version 3.7.2. Using the four algorithms (Closeness, Stress, EcCentricity, and degree), the 20 highest-ranking hub genes were recognized. Next, using the Venn diagram, common genes were extracted, including SFOS, IL-1β, CXCR4, TAC1, CDH1, and CLU (Fig. **4**).

### 3.4 LASSO Model

**Fig. (5):**
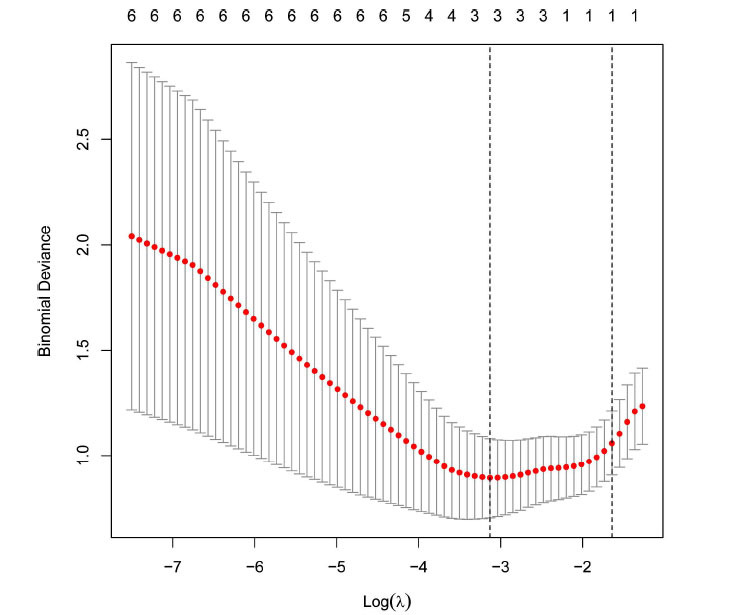
The Least Absolute Shrinkage and Selection Operator (LASSO) regression analysis of hub genes.

The construction of the LASSO model was based on the gene expression profile of the 6 common genes. Based on this model, 3 genes were identified according to their regression coefficients that were not equal to zero (Fig. **5**).

### 3.5 Verification of Gene Expression

**Fig. (6):**
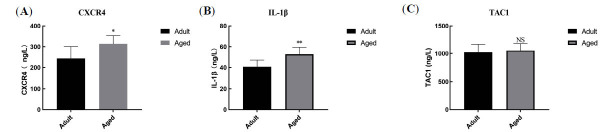
Validation of the expressions of IL-1β, CXCR4, and TAC1 in aged *versus* adult subjects. (**a-c**) IL-1β, CXCR4, and TAC1 levels. Data expressed as mean ± SD (n ≥ 10). *P < 0.05 *vs*. adult subjects, **P < 0.01 *vs*. adult subjects, NS (no significant difference) *vs*. adult subjects.

The ELISA assay was performed for the verification of the three principal gene expressions, namely, IL-1β, CXCR4, and TAC1, in adult and elderly male C57BL/6 mice. Based on our results, the IL-1β, CXCR4, and TAC1 expressions were markedly elevated in the AD group *versus* controls (Fig. **6**).

## 4 DISCUSSION

Multiple studies demonstrated associations between numerous genes and AD [32, 33]. Nevertheless, early diagnosis and *treatment* of AD remains challenging. Hence, it is both urgent and necessary to develop targeted therapeutics that can either reduce or completely relieve AD symptoms. Herein, 416 DEGs were identified as potentially significant to AD pathogenesis. Enrichment analysis of DEGs demonstrated that they particularly contributed to extracellular space, respiratory chain complex II assembly, and calcium-dependent protein binding. Furthermore, three principal AD-specific genes were revealed, namely, IL-1β, CXCR4, and TAC1. Based on our ELISA assay, we further verified that the IL-1β and CXCR4 expressions were markedly upregulated in AD patients *versus* normal subjects.

Interleukin-1 (IL-1) family is a crucial mediator of innate immunity and contributes to the inflammatory response of most cells and organs [34-39]. Aberrant signaling by members of the IL-1R family is also associated with multiple autoinflammatory and degenerative diseases [40-46]. Additionally, interleukin-1β (IL-1β), a member of the IL-1 family, is also associated with chronic inflammation [47]. Prior reports suggest that IL-1β not only participates in AD development but also serves as a key mediator of neuroinflammation [48-55]. Griffin *et al*. [56] observed that IL-1β promotes β-amyloid precursor protein generation, resulting in the synthesis and deposition of β-amyloid plaques in the brains of AD patients. Likewise, Li *et al*. [57] also reported that IL-1β is involved in tau phosphorylation, which, in turn, participates in key pathogenic processes.

Chemokines are a large family of cytokines known for their small size (8–10 kDa). It can be divided into four categories, namely, CXC, CC, C, and CX3C, based on the positioning of the initial 2 cysteines [58-63]. CXCR4, on the other hand, is vital for cellular development, hematopoiesis, organogenesis, and vascularization [64-71]. Parachikova *et al*. [72] reported that CXCR4 signaling can greatly enhance learning and memory and may be essential to AD pathogenesis. Similarly, Bonham *et al*. [73] demonstrated that the expression of microglial genes was associated functionally with CXCR4 and is often dysregulated in neurodegenerative diseases. Finally, Gavriel *et al*. [74] revealed that suppression of the CXCR4 axis can markedly augment cognitive/memory abilities, attenuate neuroinflammation, and alleviate AD symptomologies.

There are several limitations to our study. Firstly, two key genes identified in this study have not been validated in a study population. Hence, additional large-scale verification investigations are warranted to fully explore the mechanism(s) underlying AD and identify relevant genes that can be targeted for anti-AD therapy.

## CONCLUSION

In summary, the present study demonstrates that IL-1β and CXCR4 are closely related to the occurrence and progression of AD. These two genes and associated signalling are excellent potential candidates for targeted anti-AD therapy. Our study may provide novel insights into possible therapeutic targets for the treatment of AD.

## Figures and Tables

**Table 1:** Significantly enriched GO terms in AD.

**Category**	**Term ID**	**Description**	**Log P**
BP	GO:0034552	Respiratory chain complex II assembly	-4.913
	GO:0009617	Response to bacterium	-4.755
	GO:0072503	Cellular divalent inorganic cation homeostasis	-4.682
	GO:0071305	Cellular response to vitamin D	-4.229
	GO:0007586	Digestion	-3.720
	GO:0042119	Neutrophil activation	-3.661
	GO:0009629	Response to gravity	-3.615
	GO:0031623	Receptor internalization	-3.560
	GO:0043122	Regulation of I-kappa B Kinase/NF-kappa B signaling	-3.364
	GO:0014072	Response to isoquinoline alkaloid	-2.834
CC	GO:0031012	Extracellular space	-8.732
	GO:0099513	Polymeric cytoskeletal fiber	-4.250
	GO:1904724	Tertiary granule lumen	-3.831
	GO:0030667	Secretory granule membrance	-3.667
	GO:0001533	Cornified envelope	-3.311
MF	GO:0048306	Calcium-dependent protein binding	-3.511
	GO:0048018	Receptor ligand activity	-3.490
	GO:0005184	Neuropeptide hormone activity	-3.042
	GO:0004859	Phospholipase inhibitor activity	-3.001
	GO:0004859	Oxidoreductase activity, acting on NAD(P)H, oxygen as acceptor	-2.909

**Table 2:** Significantly enriched pathways in AD.

**Pathway ID**	**Name**	**Log P**
ko05150	*Staphylococcus aureus* infection	-5.829
hsa04640	Hematopoietic cell lineage	-4.048
hsa04975	Fat digestion and absorption	-3.503
hsa05332	Graft-*versus*-host disease	-3.451
hsa05340	Primary immunodeficiency	-2.695
hsa04672	Intestinal immune network for *IgA* production	-2.248

## Data Availability

The GSE48350 and GSE28146 datasets were retrieved from the GEO database.

## LIST OF ABBREVIATIONS

AD: Alzheimer’s Disease
GEO: Gene Expression Omnibus
DEGs: Differentially Expressed Genes
PPI: Protein-Protein Interaction
GO: Gene Ontology
KEGG: Kyoto Encyclopedia of Genes and Genomes
DAVID: Database for Annotation, Visualization, and Integrated Discovery
BP: Biological Process
CC: Cellular Component
MF: Molecular Function
FDR: False Discover Rate
IL-1β: Interleukin-1β
CXCR4: C-X-C Chemokine Receptor Type 4
